# The Insect database in Dokdo, Korea: An updated version in 2020

**DOI:** 10.3897/BDJ.9.e62011

**Published:** 2021-01-26

**Authors:** Jihun Ryu, Young-Kun Kim, Sang Jae Suh, Kwang Shik Choi

**Affiliations:** 1 School of Life Science, BK21 FOUR KNU Creative BioResearch Group, Kyungpook National University, Daegu, South Korea School of Life Science, BK21 FOUR KNU Creative BioResearch Group, Kyungpook National University Daegu South Korea; 2 Research Institute for Dok-do and Ulleung-do Island, Kyungpook National University, Daegu, South Korea Research Institute for Dok-do and Ulleung-do Island, Kyungpook National University Daegu South Korea; 3 School of Applied Biosciences, Kyungpook National University, Daegu, South Korea School of Applied Biosciences, Kyungpook National University Daegu South Korea; 4 Research Institute for Phylogenomics and Evolution, Kyungpook National University, Daegu, South Korea Research Institute for Phylogenomics and Evolution, Kyungpook National University Daegu South Korea

**Keywords:** biodiversity, Dokdo, insect fauna, newly-recorded species, insect database

## Abstract

**Background:**

Dokdo, a group of islands near the East Coast of South Korea, comprises 89 small islands. These volcanic islands were created by an eruption that also led to the formation of the Ulleungdo Islands (located in the East Sea), which are approximately 87.525 km away from Dokdo. Dokdo is important for geopolitical reasons; however, because of certain barriers to investigation, such as weather and time constraints, knowledge of its insect fauna is limited compared to that of Ulleungdo. Until 2017, insect fauna on Dokdo included 10 orders, 74 families, 165 species and 23 undetermined species; subsequently, from 2018 to 2019, we discovered 23 previously unrecorded species and three undetermined species via an insect survey.

**New information:**

As per our recent study, the database of insect species on Dokdo has been expanded to 10 orders, 81 families, 188 species and 23 undetermined species. This database has been registered in the Global Biodiversity Information Facility (GBIF; www.GBIF.org) and is the first record for insect fauna on Dokdo.

## Introduction

Islands are known for their ecologically- and biologically-important ecosystems. Due to geographical isolation, the movement of organisms is limited (Franks 2009) and island-like areas have low potential for both species transfer and settlement. For these reasons, as well as their small land area, islands often have low biodiversity (Mauchamp 1997). Island-like areas are also vulnerable to external intrusions, which represent a major threat to indigenous species (Kil et al. 2006). Recent developments in traffic pathways have decreased the number of isolated island ecosystems, thus allowing researchers to investigate the interactions between evolutionary and ecological processes that are responsible for island biodiversity (Gillespie et al. 2008).

The Intergovernmental Panel on Climate Change (IPCC) reports that climate change is causing sea levels and sea temperatures to rise and, if this trend continues, most coastal regions around the world will be at risk (Hong 2010, IPCC 2014). By 2100, these increases in sea level are estimated to reach at least 1 m, creating elevated flooding-related risks for large parts of the low-land island ecosystem, which could lead to significant habitat loss for many organisms worldwide (Bellard et al. 2013). The average annual sea level in Korean coastlines has been rising since 1989 and the average annual rate of sea level rise (5.67 mm/yr) around Ulleungdo has been particularly rapid (Korea Hydrographic and Oceanographic Agency 2020).

The Dokdo Islands, which are located at 37°14'26.8'' N and 131°52'10.4'' E, belong to an administrative district that includes the Ulleungdo Islands as well. The address of the Korean administrative district is 1-96 Dokdo-ri, Ulleung-eup, Ulleung-gun, Gyeongsangbuk-do, Korea. Tourists cannot stay in Dokdo for more than 30 min, although the Korean Coast Guard (KCG) and some residents can visit for longer time periods. These Islands were formed during the Pliocene Epoch by an underwater volcanic eruption that occurred between 2.5 and 4.6 million years ago (Raman et al. 2016) and subsequently promoted the formation of the Ulleungdo volcanoes via tectonic plate movement (Lee et al. 2002). Dokdo is built on sea floor that is about 2,000 m deep; it comprises two main volcanic islands and 89 small islands (Sohn 1995, Ryu et al. 2012). The nearest land area to Dokdo is Jukbyeon, Uljin-gun, Gyeongsangbuk-do, which is 217.149 km away from Dokdo and 87.525 km from Ulleungdo (Hwang and Park 2007).

Dokdo is entirely composed of volcanic rocks and its island ecosystem is relatively disconnected from the outside world; therefore, it is an important subject for island ecology and biogeography (Cultural Heritage Administration 2009). In 1982, Dokdo was designated as Natural Monument No. 336 by the Korea Cultural Heritage Administration (KCHA) to be managed by the Dokdo Natural Reserve. Dokdo is very small, with an area of 187,554 m^2^ and, because it has been protected and has inaccessible geographical features, there is extremely limited knowledge of its insect fauna. The Dokdo Islands are located at the bridge that connects Ulleungdo in Korea and the Oki Islands in Japan. Due to the fact that previous investigations of insect species on Dokdo played a vital role in characterising the biogeographic limits of these regions, the study of insect fauna on Dokdo is considered to be geographically important (Yasunaga and Duwal 2015).

Since the initial assessment of insects on Dokdo by Jolivet in 1974, many researchers have conducted follow-up studies and, by 2017, 10 orders, 74 families and 165 species of insects have been identified (Jolivet 1974, Yoon 1978, Lee and Kwon 1981, Kwon et al. 1996, An 2000, Korean Ministry of Environment 2001, Ulleung Research Institute of Gyeongju University 2004, Park and Suh 2005, Kim and Yeom 2006, An 2008, Kim et al. 2009, Lee et al. 2009, Park et al. 2010, Park et al. 2011, Park et al. 2013, Daegu Regional Environmental Office 2012, Daegu Regional Environmental Office 2016, Choi et al. 2015, Park et al. 2017, Hwang et al. 2017). Here, we report 23 newly-identified and unrecorded species and three undetermined species on the Island and provide an updated database.

## Sampling methods

### Study extent

**Figure 1.**
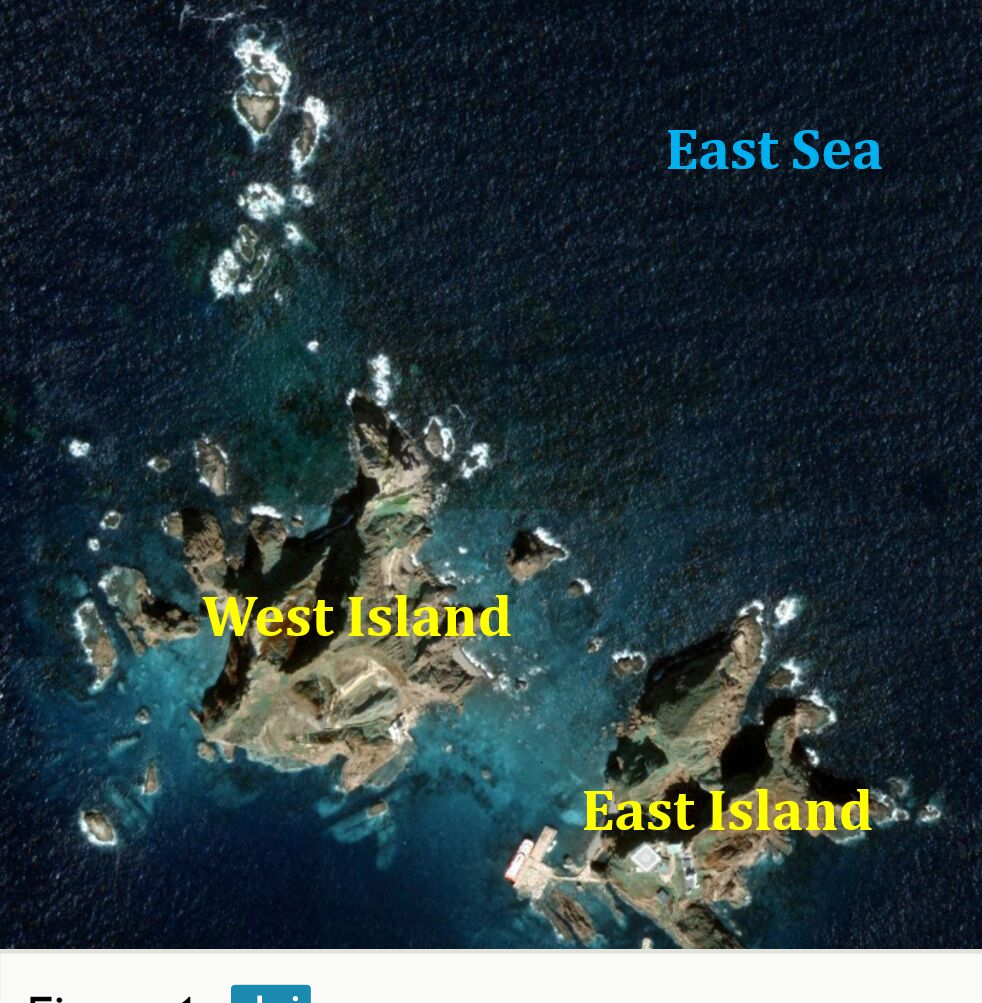
Map of Dokdo: East (Dongdo) and West (Seodo) Islands.

Between September 2017 and September 2018, we collected samples from Dokdo four times, once each at Anyongbok-gil, Ulleung-eup, Ulleung-gun and Gyeongsangbuk-do, Korea (131°52'03.2''E, 37°14'27.2'N), using sweeping, beating, brandishing, black light traps and pitfall traps. The survey was divided into East (Dongdo) and West (Seodo) using terrain isolation (Fig. 1).

### Sampling description

In Dongdo, we performed sample collection along the slope leading from the marina, through the KCG facility and then to the old marina. In Seodo, we performed sample collection along a very steep slope leading to the fishermen's dormitory. Seodo has less vegetation distribution compared to Dongdo.

Collected specimen samples were stored in 70% ethanol in conical tubes. They were then transferred to the Animal Systematics and Taxonomy Laboratory and Pest Control Laboratory at Kyungpook National University, Korea. The samples were identified using the national species list of Korea and other references (National Institute of Biological Resources 2019, Chérot and Malipatil 2016, Yasunaga 2017, Göllner-Scheiding 1976, Slater and Zheng 1984, Siwi and Doesburg 1984, Malenovský 2013, ZHANG et al. 2013, Viraktamath 1979, Kim 2012, YOO et al. 2008, Yang et al. 2014, Kurahashi and Samerjai 2018, Suh and Kwon 2017a, Suh and Kwon 2017b, Bourquia et al. 2019, Vane-Wright and Hughes 2007, Ang and Meier 2010).

**Database update**: We created a new checklist by adding 23 newly-confirmed insect species with reference to the previous reports and compiled it into a database. The data have been registered in the Global Biodiversity Information Facility (GBIF).

## Geographic coverage

### Description

The survey was divided into Dongdo and Seodo.

### Coordinates

 and 37-14 Latitude Latitude; and 131-52 and 131-51 longitude Longitude.

## Taxonomic coverage

### Taxa included

**Table:** 

Rank	Scientific Name	Common Name
kingdom	Animalia	Animals
phylum	Arthropoda	Arthropods
class	Insecta	Insects
order	Blattodea	
family	Ectobiidae	
order	Coleoptera	
family	Aphodiidae	
family	Carabidae	
family	Chrysomelidae	
family	Coccinellidae	
family	Curculionidae	
family	Elateridae	
family	Endomychidae	
family	Hydrophilidae	
family	Latridiidae	
family	Mordellidae	
family	Nitidulidae	
family	Oedemeridae	
family	Scirtidae	
family	Staphylinidae	
family	Tenebrionidae	
order	Dermaptera	
family	Anisolabididae	
family	Forficulidae	
order	Diptera	
family	Anthomyiidae	
family	Calliphoridae	
family	Ceratopogonidae	
family	Chironomidae	
family	Chloropidae	
family	Coelopidae	
family	Culicidae	
family	Drosophilidae	
family	Muscidae	
family	Phoridae	
family	Psychodidae	
family	Rhiniidae	
family	Sarcophagidae	
family	Scathophagidae	
family	Sepsidae	
family	Syrphidae	
family	Tephritidae	
family	Tipulidae	
order	Hemiptera	
family	Alydidae	
family	Anthocoridae	
family	Aphididae	
family	Cicadellidae	
family	Cydnidae	
family	Delphacidae	
family	Lygaeidae	
family	Miridae	
family	Nabidae	
family	Pentatomidae	
family	Piesmatidae	
family	Rhopalidae	
family	Rhyparochromidae	
family	Scutelleridae	
family	Tingidae	
family	Triozidae	
order	Hymenoptera	
family	Bethylidae	
family	Braconidae	
family	Chalcididae	
family	Eupelmidae	
family	Formicidae	
family	Ichneumonidae	
family	Pteromalidae	
order	Lepidoptera	
family	Crambidae	
family	Erebidae	
family	Geometridae	
family	Hesperiidae	
family	Lycaenidae	
family	Noctuidae	
family	Nymphalidae	
family	Papilionidae	
family	Plutellidae	
family	Pyralidae	
family	Sphingidae	
family	Tortricidae	
family	Yponomeutidae	
order	Neuroptera	
family	Chrysopidae	
family	Hemerobiidae	
family	Sisyridae	
order	Odonata	
family	Aeshnidae	
family	Coenagrionidae	
family	Libellulidae	
order	Orthoptera	
family	Gryllacrididae	
family	Gryllidae	
family	Mogoplistidae	

## Usage licence

### Usage licence

Creative Commons Public Domain Waiver (CC-Zero)

## Data resources

### Data package title

dokdo insect list, 1974-2019

### Resource link

https://doi.org/10.15468/h684as

### Number of data sets

1

### Data set 1.

#### Data set name

dokdo_insect_list_1974-2019

#### Data format

CSV.

#### Number of columns

24

#### Download URL

https://www.gbif.org/dataset/eb47a0de-862f-44c0-be42-0e300abaaab4

#### 

**Data set 1.** 

Column label	Column description
occurrenceID	Unique identifier of the occurrence
basisOfRecord	State of the recorded specimen
eventDate	Date of the data registration
institutionCode	Abbreviation of the institution having custody of the object
scientificName	Full scientific name
ScientificNameAuthorship	The authorship information for the scientificName, formatted according to the conventions of the applicable nomenclaturalCode
collectionCode	Abbreviation of specimen or database
decimalLatitude	Geographic latitude of the collection site
decimalLongitude	Geographic longitude of the collection site
coordinateUncertaintyInMetres	The horizontal distance (in metres) from the given decimalLatitude and decimalLongitude describing the smallest circle containing the whole of the Location
countryCode	Country code
Identification Date	Date for identifying the specimen
Identified by	Identifier for the specimen
stateProvince	Province in which the specimen was collected
county	County in which the specimen was collected
locality	Locality in which the specimen was collected
catalogNumber	Specimen number for occurrence
vernacularName	Common or vernacular name in Korea
kingdom	kingdom name
phylum	phylum name
class	class name
order	order name
family	family name
genus	genus name

## Additional information

### Results and Discussion

In this study, we first created an initial database, based on the results of surveys of insects on Dokdo from 1974 to 2017. This checklist confirmed the identification of 10 orders, 74 families, 165 species and 23 undetermined species of insects distributed on the Island.

**Figure 2.**
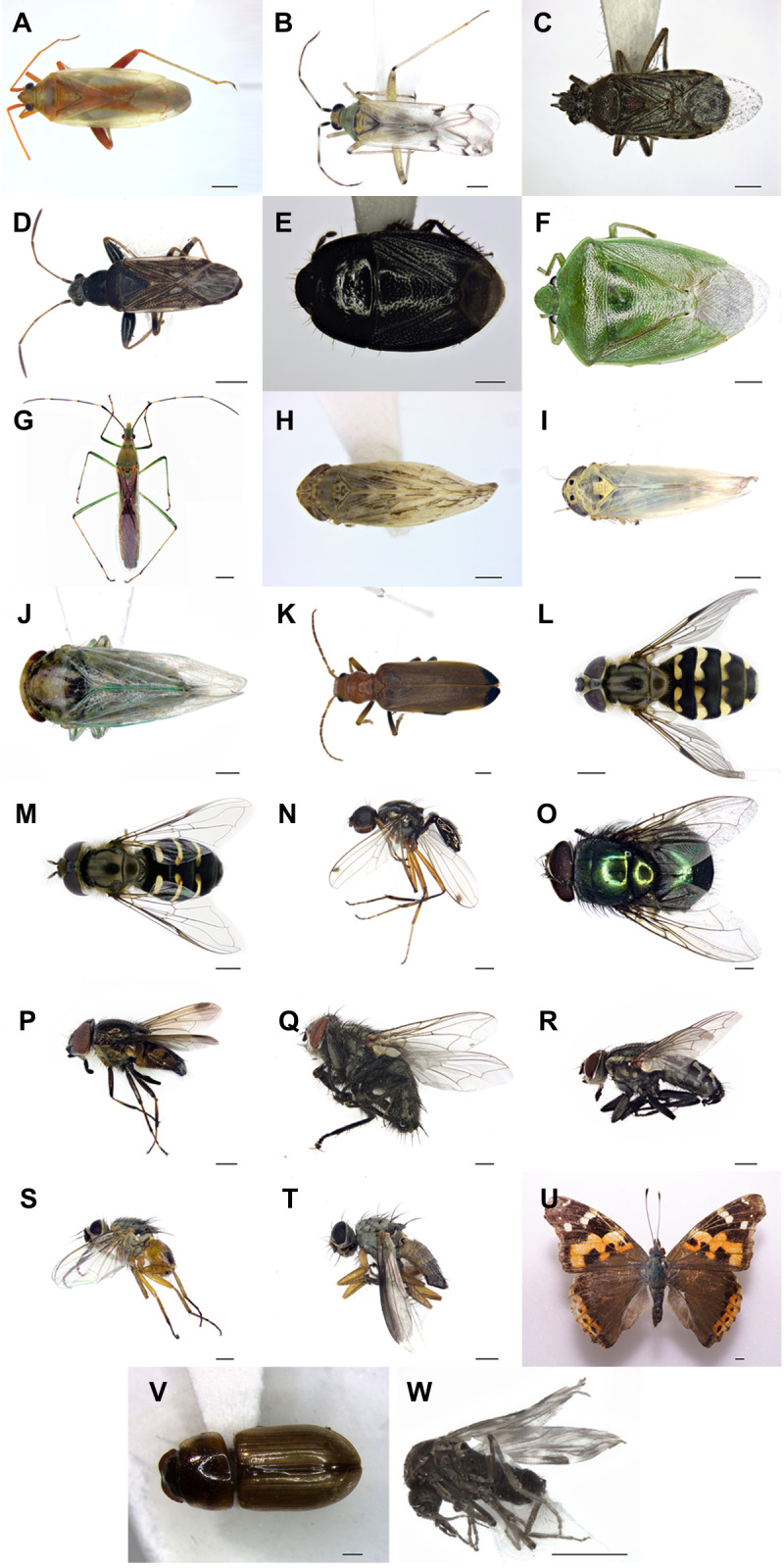
Newly-recorded insect species on Dokdo Island. **A.** *Creontiades coloripes*; **B.**; *Nesidiocoris tenuis*; **C.** *Liorhyssus hyalinus*; **D.** *Horridipamera inconspicua*; **E.** *Geotomus convexus*; **F.** *Glaucias subpunctatus*; **G.** *Leptocorisa chinensis*; **H.** *Recilia coronifera*; **I.** *Macrosteles striifrons*; **J.** *Batracomorphus diminuta*; **K.** *Nacerdes melanura*; **L.** *Dasysyrphus bilineatus*; **M.** *Scaeva komabensis*; **N.** *Sepsis monostigma*; **O.** *Lucilia porphyrina*; **P.** *Stomorhina obsoleta*; **Q.** *Sarcophaga brevicornis*; **R.** *Sarcophaga peregrina*; **S.** *Atherigona oryzae*; **T.** *Orchisia costata*; **U.** *Vanessa indica*; **V.** *Aphodius urostigma*; **W.** *Culicoides circumscriptus*. Scale bars: F, G, L, M, R, U = 2.0 mm; A, C, D, K, O, P, Q = 1.0 mm; B, E, H, I, J, N, S, T, V, W = 0.5 mm.

Through our recent sampling, we collected and identified 23 previously unrecorded insect species in Dokdo. Of these species, ten are in the Order Hemiptera (*Leptocorisa chinensis*, *Batracomorphus diminuta*, *Macrosteles striifrons*, *Recilia coronifera*, *Geotomus convexus*, *Creontiades coloripes*, *Nesidiocoris tenuis*, *Glaucias subpunctatus*, *Liorhyssus hyalinus* and *Horridipamera inconspicua*); two are in the Order Coleoptera (*Aphodius urostigma* and *Nacerdes melanura*); ten are in the Order Diptera (*Dasysyrphus bilineatus*, *Scaeva komabensis*, *Sepsis monostigma*, *Lucilia porphyrina*, *Stomorhina obsoleta*, *Sarcophaga brevicornis*, *Sarcophaga peregrina*, *Atherigona oryzae*, *Orchisia costata* and *Culicoides circumscriptus*); and one is in the Order Lepidoptera (*Vanessa indica*) (Fig. 2).

In addition to the 23 previously unrecorded species on Dokdo, three undetermined species were identified to the genus level: two *Empoasca* species in the Family Cicadellidae and one *Sisyra* species in the Family Sisyridae. Furthermore, five of the 23 previously unrecorded species and one of the three unidentified species belong to new families, adding a total of six newly-added families to the Dokdo Insect Database. Finally, we generated an updated database of insect fauna on Dokdo that contains 10 orders, 81 families, 188 species and 23 unidentified species (Table 1).

Amongst the 23 previously unrecorded species on Dokdo, *B. diminuta* was first discovered in Korea and the remainder of this species has been recorded in the Korean Peninsula. In addition, *C. circumscriptus*, a blood-sucking insect known for being extremely annoying to the KCG and residents of Dokdo, has been identified for the first time in Dokdo, using a black light trap with dry ice.

The 23 undetermined species that have been identified up to the genus stage are classified as unrecorded or new species in Korea. Due to geographical characteristics, the study of these insects is considered to be very important for understanding the biodiversity of Dokdo Island.

### Conflicts of interest

The authors declare that there are no conflicts of interest.

## Figures and Tables

**Table 1.** The updated database of insect species on Dokdo, based on data collected from 1974 to 2019.

Order																																																																																																																								Family																																																																																Scientific Name	Newly recorded species	Undetermined name
Blattodea																																																																																																																								Ectobiidae																																																																																*Blattella nipponica* Asahina, 1963		
Coleoptera																																																																																																																								Aphodiidae																																																																																*Aphodius urostigma* Harold, 1862	O	
Coleoptera																																																																																																																								Carabidae																																																																																*Anisodactylus signatus* (Panzer, 1796)		
Coleoptera																																																																																																																								Carabidae																																																																																*Anisodactylus tricuspidatus* A.Morawitz, 1863		
Coleoptera																																																																																																																								Carabidae																																																																																*Dolichus halensis* (Schaller, 1783)		
Coleoptera																																																																																																																								Carabidae																																																																																*Harpalus jureceki* (Jedlicka, 1928)		
Coleoptera																																																																																																																								Carabidae																																																																																*Harpalus sinicus* Hope, 1845		
Coleoptera																																																																																																																								Carabidae																																																																																*Stenolophus difficilis* (Hope, 1845)		
Coleoptera																																																																																																																								Chrysomelidae																																																																																*Callosobruchus chinensis* (Linnaeus, 1758)		
Coleoptera																																																																																																																								Chrysomelidae																																																																																*Cassida nebulosa* Linnaeus, 1758		
Coleoptera																																																																																																																								Chrysomelidae																																																																																*Cassida piperata* Hope, 1842		
Coleoptera																																																																																																																								Chrysomelidae																																																																																*Longitarsus succineus* (Foudras, 1859)		
Coleoptera																																																																																																																								Chrysomelidae																																																																																*Psylliodes punctifrons* Baly, 1874		
Coleoptera																																																																																																																								Chrysomelidae																																																																																*Thlaspida biramosa* (Boheman, 1855)		
Coleoptera																																																																																																																								Coccinellidae																																																																																*Coccinella septempunctata* Linnaeus, 1758		
Coleoptera																																																																																																																								Coccinellidae																																																																																*Harmonia axyridis* (Pallas, 1773)		
Coleoptera																																																																																																																								Coccinellidae																																																																																*Harmonia yedoensis* (Takizawa, 1917)		
Coleoptera																																																																																																																								Coccinellidae																																																																																*Propylea japonica* (Thunberg, 1781)		
Coleoptera																																																																																																																								Coccinellidae																																																																																*Scymnus babai* Sasaji, 1971		
Coleoptera																																																																																																																								Coccinellidae																																																																																*Scymnus ferrugatus* (Moll, 1785)		
Coleoptera																																																																																																																								Coccinellidae																																																																																*Scymnus* sp. Kugelann, 1794		O
Coleoptera																																																																																																																								Curculionidae																																																																																*Auleutes* sp. Dietz, 1896		O
Coleoptera																																																																																																																								Curculionidae																																																																																*Cosmobaris scolopacea* Germar, 1819		
Coleoptera																																																																																																																								Curculionidae																																																																																*Ceutorhynchus albosuturalis* (Roelofs, 1875)		
Coleoptera																																																																																																																								Curculionidae																																																																																*Dermestes tessellatocollis* Motschulsky, 1860		
Coleoptera																																																																																																																								Curculionidae																																																																																*Rhinoncus cribricollis* Hustache, 1916		
Coleoptera																																																																																																																								Curculionidae																																																																																*Rhinoncus jakovlevi* Faust, 1893		
Coleoptera																																																																																																																								Curculionidae																																																																																*Scepticus insularis* Roelofs, 1873		
Coleoptera																																																																																																																								Curculionidae																																																																																*Scepticus uniformis* Kono, 1930		
Coleoptera																																																																																																																								Curculionidae																																																																																*Sitona lineatus* (Linnaeus, 1758)		
Coleoptera																																																																																																																								Elateridae																																																																																*Agrypnus miyamotoi* (Nakane & Kishii, 1955)		
Coleoptera																																																																																																																								Elateridae																																																																																*Melanotus castanipes* (Paykull, 1800)		
Coleoptera																																																																																																																								Elateridae																																																																																*Melanotus cete* Candèze, 1860		
Coleoptera																																																																																																																								Elateridae																																																																																*Pectocera fortunei* Candéze, 1873		
Coleoptera																																																																																																																								Endomychidae																																																																																*Ancylopus melanocephalus* (Olivier, 1808)		
Coleoptera																																																																																																																								Endomychidae																																																																																*Ancylopus pictus asiaticus* (Strohecker, 1972)		
Coleoptera																																																																																																																								Hydrophilidae																																																																																*Hydrophilus acuminatus* Motschulsky, 1854		
Coleoptera																																																																																																																								Latridiidae																																																																																*Cortinicara gibbosa* (Herbst, 1793)		
Coleoptera																																																																																																																								Latridiidae																																																																																*Stephostethus chinensis* (Reitter, 1877)		
Coleoptera																																																																																																																								Mordellidae																																																																																*Mordella* sp. Linnaeus, 1758		O
Coleoptera																																																																																																																								Mordellidae																																																																																*Mordella tokejii* Nomura, 1958		
Coleoptera																																																																																																																								Nitidulidae																																																																																*Omosita colon* (Linnaeus, 1758)		
Coleoptera																																																																																																																								Nitidulidae																																																																																*Omosita japonica* Reitter, 1874		
Coleoptera																																																																																																																								Oedemeridae																																																																																*Nacerdes melanura* (Linnaeus, 1758)	O	
Coleoptera																																																																																																																								Scirtidae																																																																																*Cyphon* sp. Paykull, 1799		O
Coleoptera																																																																																																																								Staphylinidae																																																																																*Aleochara fucicola* Sharp, 1874		
Coleoptera																																																																																																																								Staphylinidae																																																																																*Atheta* sp. Thomson, 1858		O
Coleoptera																																																																																																																								Staphylinidae																																																																																*Atheta tokiokai* (K.Sawada, 1971)		
Coleoptera																																																																																																																								Staphylinidae																																																																																*Cafius histrio* (Sharp, 1874)		
Coleoptera																																																																																																																								Staphylinidae																																																																																*Neobisnius* sp. Ganglbauer, 1895		O
Coleoptera																																																																																																																								Staphylinidae																																																																																*Paederus fuscipes* Curtis, 1826		
Coleoptera																																																																																																																								Tenebrionidae																																																																																*Gonocephalum coenosum* Kaszab, 1952		
Coleoptera																																																																																																																								Tenebrionidae																																																																																*Gonocephalum coriaceum* Motschulsky, 1857		
Dermaptera																																																																																																																								Anisolabididae																																																																																*Anisolabis maritima* (Bonelli, 1832)		
Dermaptera																																																																																																																								Anisolabididae																																																																																*Euborellia annulipes* (H.F.Lucas, 1847)		
Dermaptera																																																																																																																								Forficulidae																																																																																*Forficula scudderi* de Bormans, 1880		
Diptera																																																																																																																								Anthomyiidae																																																																																*Delia platura* (Meigen, 1826)		
Diptera																																																																																																																								Anthomyiidae																																																																																*Fucellia apicalis* Kertesz, 1908		
Diptera																																																																																																																								Anthomyiidae																																																																																*Fucellia boninensis* Snyder, 1965		
Diptera																																																																																																																								Anthomyiidae																																																																																*Pegomya cunicularia* (Rondani, 1866)		
Diptera																																																																																																																								Calliphoridae																																																																																*Calliphora nigribarbis* Smellen van Vollenhoven, 1863		
Diptera																																																																																																																								Calliphoridae																																																																																*Hemipyrellia ligurriens* (Wiedemann, 1830)		
Diptera																																																																																																																								Calliphoridae																																																																																*Lucilia illustris* (Meigen, 1826)		
Diptera																																																																																																																								Calliphoridae																																																																																*Lucilia porphyrina* (Walker, 1856)	O	
Diptera																																																																																																																								Calliphoridae																																																																																*Lucilia sericata* (Meigen, 1826)		
Diptera																																																																																																																								Ceratopogonidae																																																																																*Culicoides circumscriptus* Kieffer, 1918	O	
Diptera																																																																																																																								Chironomidae																																																																																*Polypedilum* sp. Kieffer, 1912		O
Diptera																																																																																																																								Chloropidae																																																																																*Thaumatomyia notata* (Meigen, 1830)		
Diptera																																																																																																																								Coelopidae																																																																																*Coelopa frigida* (Fabricius, 1805)		
Diptera																																																																																																																								Culicidae																																																																																*Culex orientalis* Edwards, 1921		
Diptera																																																																																																																								Culicidae																																																																																*Ochlerotatus togoi* (Theobald, 1907)		
Diptera																																																																																																																								Drosophilidae																																																																																*Drosophila* sp. Fallén, 1823		O
Diptera																																																																																																																								Muscidae																																																																																*Atherigona oryzae* Malloch, 1925	O	
Diptera																																																																																																																								Muscidae																																																																																*Musca bezzii* Patton & Cragg, 1913		
Diptera																																																																																																																								Muscidae																																																																																*Musca hervei* Villeneuve, 1922		
Diptera																																																																																																																								Muscidae																																																																																*Orchisia costata* (Meigen, 1826)	O	
Diptera																																																																																																																								Phoridae																																																																																*Megaselia spiracularis* Schmitz, 1938		
Diptera																																																																																																																								Psychodidae																																																																																*Psychoda alternata* Say, 1824		
Diptera																																																																																																																								Psychodidae																																																																																*Tinearia alternata* (Say, 1824)		
Diptera																																																																																																																								Rhiniidae																																																																																*Stomorhina obsoleta* Wiedemann, 1830	O	
Diptera																																																																																																																								Sarcophagidae																																																																																*Sarcophaga brevicornis* Ho, 1934	O	
Diptera																																																																																																																								Sarcophagidae																																																																																*Sarcophaga melanura* Meigen, 1826		
Diptera																																																																																																																								Sarcophagidae																																																																																*Sarcophaga peregrina* (Robineau-Desvoidy, 1830)	O	
Diptera																																																																																																																								Scathophagidae																																																																																*Scathophaga stercoraria* (Linnaeus, 1758)		
Diptera																																																																																																																								Sepsidae																																																																																*Sepsis monostigma* Thomson, 1869	O	
Diptera																																																																																																																								Syrphidae																																																																																*Allobaccha apicalis* (Loew, 1858)		
Diptera																																																																																																																								Syrphidae																																																																																*Allograpta javana* (Wiedemann, 1824)		
Diptera																																																																																																																								Syrphidae																																																																																*Betasyrphus serarius* (Wiedemann, 1830)		
Diptera																																																																																																																								Syrphidae																																																																																*Dasysyrphus bilineatus* (Matsumura, 1917)	O	
Diptera																																																																																																																								Syrphidae																																																																																*Episyrphus balteatus* (De Geer, 1776)		
Diptera																																																																																																																								Syrphidae																																																																																*Eristalis cerealis* Fabricius, 1805		
Diptera																																																																																																																								Syrphidae																																																																																*Eristalis tenax* (Linnaeus, 1758)		
Diptera																																																																																																																								Syrphidae																																																																																*Metasyrphus corollae* (Fabricius, 1794)		
Diptera																																																																																																																								Syrphidae																																																																																*Metasyrphus nitens* (Zetterstedt, 1843)		
Diptera																																																																																																																								Syrphidae																																																																																*Scaeva komabensis* (Matsumura, 1918)	O	
Diptera																																																																																																																								Syrphidae																																																																																*Melanostoma mellinum* (Linnaeus, 1758)		
Diptera																																																																																																																								Syrphidae																																																																																*Sphaerophoria menthastri* (Linnaeus, 1758)		
Diptera																																																																																																																								Syrphidae																																																																																*Xanthandrus comtus* (Harris, 1780)		
Diptera																																																																																																																								Tephritidae																																																																																*Campiglossa sada* (Dirlbek & Dirlbekova, 1974)		
Diptera																																																																																																																								Tephritidae																																																																																*Campiglossa* sp. Rondani, 1870		O
Diptera																																																																																																																								Tephritidae																																																																																*Ensina sonchi* (Linnaeus, 1767)		
Diptera																																																																																																																								Tephritidae																																																																																*Trupanea convergens* (Hering, 1936)		
Diptera																																																																																																																								Tipulidae																																																																																*Tipula* sp. Linnaeus, 1758		O
Hemiptera																																																																																																																								Alydidae																																																																																*Leptocorisa chinensis* Dallas, 1852	O	
Hemiptera																																																																																																																								Anthocoridae																																																																																*Orius sauteri* (Poppius, 1909)		
Hemiptera																																																																																																																								Anthocoridae																																																																																*Orius* sp. Distant, 1904		O
Hemiptera																																																																																																																								Aphididae																																																																																*Aphis nerii* Fonscolombe, 1841		
Hemiptera																																																																																																																								Aphididae																																																																																*Aphis rumicis* Linnaeus, 1758		
Hemiptera																																																																																																																								Cicadellidae																																																																																*Balclutha rubrinervis* Matsumura, 1902		
Hemiptera																																																																																																																								Cicadellidae																																																																																*Batracomorphus diminuta* Matsumura, 1912	O	
Hemiptera																																																																																																																								Cicadellidae																																																																																*Empoasca* sp.1 Walsh, 1862		O
Hemiptera																																																																																																																								Cicadellidae																																																																																*Empoasca* sp.2 Walsh, 1862		O
Hemiptera																																																																																																																								Cicadellidae																																																																																*Hishimonus sellatus* Uhler, 1896		
Hemiptera																																																																																																																								Cicadellidae																																																																																*Laburrus impictifrons* Boheman, 1852		
Hemiptera																																																																																																																								Cicadellidae																																																																																*Macrosteles striifrons* Anufriev, 1968	O	
Hemiptera																																																																																																																								Cicadellidae																																																																																*Maiestas oryzae* Matsumura, 1902		
Hemiptera																																																																																																																								Cicadellidae																																																																																*Psammotettix striatus* Linnaeus, 1758		
Hemiptera																																																																																																																								Cicadellidae																																																																																*Recilia coronifera* (Marshall, 1866)	O	
Hemiptera																																																																																																																								Cydnidae																																																																																*Geotomus pygmaeus* (Dallas, 1851)		
Hemiptera																																																																																																																								Cydnidae																																																																																*Geotomus convexus* Hsiao, 1977	O	
Hemiptera																																																																																																																								Delphacidae																																																																																*Laodelphax striatellus* (Fallén, 1826)		
Hemiptera																																																																																																																								Delphacidae																																																																																*Sogatella furcifera* (Horváth, 1899)		
Hemiptera																																																																																																																								Delphacidae																																																																																*Sogatella kolophon* (Kirkaldy, 1907)		
Hemiptera																																																																																																																								Delphacidae																																																																																*Unkanodes sapporonus* (Matsumura, 1935)		
Hemiptera																																																																																																																								Lygaeidae																																																																																*Nysius plebeius* Distant & W.L., 1883		
Hemiptera																																																																																																																								Miridae																																																																																*Campylomma lividicornis* Reuter, 1912		
Hemiptera																																																																																																																								Miridae																																																																																*Campylomma* sp. Reuter, 1878		O
Hemiptera																																																																																																																								Miridae																																																																																*Creontiades coloripes* Hsiao & Meng, 1963	O	
Hemiptera																																																																																																																								Miridae																																																																																*Nesidiocoris tenuis* (Reuter, 1895)	O	
Hemiptera																																																																																																																								Miridae																																																																																*Orthotylus flavosparsus* (C.Sahlberg, 1841)		
Hemiptera																																																																																																																								Miridae																																																																																*Trigonotylus caelestialium* (Kirkaldy, 1902)		
Hemiptera																																																																																																																								Nabidae																																																																																*Prostemma hilgendorfii* Stein, 1878		
Hemiptera																																																																																																																								Pentatomidae																																																																																*Glaucias subpunctatus* (Walker, 1867)	O	
Hemiptera																																																																																																																								Pentatomidae																																																																																*Nezara antennata* Scott, 1874		
Hemiptera																																																																																																																								Piesmatidae																																																																																*Piesma capitatum* (Wolff, 1804)		
Hemiptera																																																																																																																								Piesmatidae																																																																																*Piesma maculatum* (Laporte, 1833)		
Hemiptera																																																																																																																								Rhopalidae																																																																																*Liorhyssus hyalinus* (Fabricius, 1794)	O	
Hemiptera																																																																																																																								Rhyparochromidae																																																																																*Horridipamera inconspicua* Dallas, 1852	O	
Hemiptera																																																																																																																								Rhyparochromidae																																																																																*Paradieuches dissimilis* (Distant & W.L., 1883)		
Hemiptera																																																																																																																								Rhyparochromidae																																																																																*Stigmatonotum rufipes* (V.Motschulsky, 1866)		
Hemiptera																																																																																																																								Scutelleridae																																																																																*Cantao ocellatus* (Thunberg, 1784)		
Hemiptera																																																																																																																								Tingidae																																																																																*Cantacader lethierryi* Scott, 1874		
Hemiptera																																																																																																																								Triozidae																																																																																*Trioza chenopodi* Reuter, 1876		
Hymenoptera																																																																																																																								Bethylidae																																																																																*Acrepyris minutus* Yasumatsu, 1955		
Hymenoptera																																																																																																																								Braconidae																																																																																*Apanteles* sp. Förster, 1862		O
Hymenoptera																																																																																																																								Braconidae																																																																																*Cotesia* sp.1 Cameron, 1891		O
Hymenoptera																																																																																																																								Braconidae																																																																																*Cotesia* sp.2 Cameron, 1891		O
Hymenoptera																																																																																																																								Braconidae																																																																																*Deuterixys* sp. Mason, 1981		O
Hymenoptera																																																																																																																								Braconidae																																																																																*Lysiphlebus* sp. Förster, 1862		O
Hymenoptera																																																																																																																								Chalcididae																																																																																*Brachymeria femorata* (Panzer, 1801)		
Hymenoptera																																																																																																																								Chalcididae																																																																																*Brachymeria minuta* (Linnaeus, 1767)		
Hymenoptera																																																																																																																								Eupelmidae																																																																																*Eupelmus australiensis* (Girault, 1913)		
Hymenoptera																																																																																																																								Eupelmidae																																																																																*Eupelmus* sp. Dalman, 1820		O
Hymenoptera																																																																																																																								Formicidae																																																																																*Hypoponera nippona* (Santschi, 1937)		
Hymenoptera																																																																																																																								Formicidae																																																																																*Lasius meridionalis* (Bondroit, 1920)		
Hymenoptera																																																																																																																								Formicidae																																																																																*Monomorium floricola* (Jerdon, 1851)		
Hymenoptera																																																																																																																								Formicidae																																																																																*Monomorium intrudens* Smith, 1874		
Hymenoptera																																																																																																																								Formicidae																																																																																*Myrmecina graminicola nipponica* Wheeler, 1906		
Hymenoptera																																																																																																																								Formicidae																																																																																*Pachycondyla chinensis* (Emery, 1895)		
Hymenoptera																																																																																																																								Formicidae																																																																																*Pheidole fervida* Smith, 1874		
Hymenoptera																																																																																																																								Formicidae																																																																																*Ponera japonica* Wheeler, 1906		
Hymenoptera																																																																																																																								Formicidae																																																																																*Pristomyrmex pungens* Mayr, 1866		
Hymenoptera																																																																																																																								Formicidae																																																																																*Solenopsis japonica* Wheeler, 1928		
Hymenoptera																																																																																																																								Formicidae																																																																																*Strumigenys lewisi* Cameron, 1886		
Hymenoptera																																																																																																																								Formicidae																																																																																*Tetramorium caespitum* (Linnaeus, 1758)		
Hymenoptera																																																																																																																								Ichneumonidae																																																																																*Homotropus* sp. Förster, 1869		O
Hymenoptera																																																																																																																								Pteromalidae																																																																																*Halticoptera circulus* (Walker, 1833)		
Lepidoptera																																																																																																																								Crambidae																																																																																*Cnaphalocrocis medinalis* Guenée, 1854		
Lepidoptera																																																																																																																								Crambidae																																																																																*Diaphania indica* (Saunders, 1851)		
Lepidoptera																																																																																																																								Crambidae																																																																																*Maruca vitrata* (Fabricius, 1787)		
Lepidoptera																																																																																																																								Crambidae																																																																																*Palpita nigropunctalis* Bremer, 1864		
Lepidoptera																																																																																																																								Crambidae																																																																																*Spoladea recurvalis* (Fabricius, 1775)		
Lepidoptera																																																																																																																								Erebidae																																																																																*Catocala dula* Bremer, 1861		
Lepidoptera																																																																																																																								Geometridae																																																																																*Odontopera arida* Butler, 1878		
Lepidoptera																																																																																																																								Geometridae																																																																																*Scopula ignobilis* Warren, 1901		
Lepidoptera																																																																																																																								Hesperiidae																																																																																*Parnara guttatus* Bremer & Grey, 1853		
Lepidoptera																																																																																																																								Lycaenidae																																																																																*Arhopala bazalus* (Hewitson, 1862)		
Lepidoptera																																																																																																																								Lycaenidae																																																																																*Pseudozizeeria maha* (Kollar, 1844)		
Lepidoptera																																																																																																																								Noctuidae																																																																																*Agrotis ipsilon* (Hufnagel, 1766)		
Lepidoptera																																																																																																																								Noctuidae																																																																																*Arcte coerula* Guenée, 1852		
Lepidoptera																																																																																																																								Noctuidae																																																																																*Callopistria argyrosticta* Butler, 1881		
Lepidoptera																																																																																																																								Noctuidae																																																																																*Cosmia achatina* Butler, 1879		
Lepidoptera																																																																																																																								Noctuidae																																																																																*Daddala lucilla* Butler, 1881		
Lepidoptera																																																																																																																								Noctuidae																																																																																*Diarsia canescens* Butler, 1878		
Lepidoptera																																																																																																																								Noctuidae																																																																																*Thyas juno* (Dalman, 1823)		
Lepidoptera																																																																																																																								Noctuidae																																																																																*Macdunnoughia confusa* (Stephens, 1850)		
Lepidoptera																																																																																																																								Noctuidae																																																																																*Mythimna separata* (Walker, 1865)		
Lepidoptera																																																																																																																								Nymphalidae																																																																																*Cynthia cardui* Linnaeus, 1758		
Lepidoptera																																																																																																																								Nymphalidae																																																																																*Vanessa indica* Herbst, 1794	O	
Lepidoptera																																																																																																																								Papilionidae																																																																																*Papilio xuthus* Linnaeus, 1767		
Lepidoptera																																																																																																																								Plutellidae																																																																																*Plutella xylostella* (Linnaeus, 1758)		
Lepidoptera																																																																																																																								Pyralidae																																																																																*Oncocera semirubella* (Scopoli, 1763)		
Lepidoptera																																																																																																																								Sphingidae																																																																																*Macroglossum stellatarum* (Linnaeus, 1758)		
Lepidoptera																																																																																																																								Tortricidae																																																																																*Adoxophyes orana* (Fischer v.Roslerstamm, 1834)		
Lepidoptera																																																																																																																								Tortricidae																																																																																*Archips oporana* (Linnaeus, 1758)		
Lepidoptera																																																																																																																								Tortricidae																																																																																*Cochylidia contumescens* Meyrick, 1931		
Lepidoptera																																																																																																																								Tortricidae																																																																																*Cochylidia richteriana* (Fischer v.Roslerstamm, 1837)		
Lepidoptera																																																																																																																								Tortricidae																																																																																*Tortrix sinapina* Butler, 1879		
Lepidoptera																																																																																																																								Yponomeutidae																																																																																*Yponomeuta meguronis* Matsumura, 1931		
Neuroptera																																																																																																																								Chrysopidae																																																																																*Chrysopa pallens* (Rambur, 1838)		
Neuroptera																																																																																																																								Hemerobiidae																																																																																*Hemerobius humulinus* Linnaeus, 1758		
Neuroptera																																																																																																																								Sisyridae																																																																																*Sisyra* sp. Burmeister, 1839		O
Odonata																																																																																																																								Aeshnidae																																																																																*Anax parthenope* Selys, 1839		
Odonata																																																																																																																								Coenagrionidae																																																																																*Ischnura asiatica* Brauer, 1865		
Odonata																																																																																																																								Libellulidae																																																																																*Pantala flavescens* Fabricius, 1798		
Odonata																																																																																																																								Libellulidae																																																																																*Rhyothemis fuliginosa* Selys, 1883		
Odonata																																																																																																																								Libellulidae																																																																																*Sympetrum darwinianum* Selys, 1883		
Orthoptera																																																																																																																								Gryllacrididae																																																																																*Nippancistroger* sp. Griffini, 1913		O
Orthoptera																																																																																																																								Gryllidae																																																																																*Teleogryllus emma* (Ohmachi & I.Matsuura, 1951)		
Orthoptera																																																																																																																								Gryllidae																																																																																*Velarifictorus aspersus* (F.Walker, 1869)		
Orthoptera																																																																																																																								Mogoplistidae																																																																																*Ornebius kanetataki* (S.Matsumura, 1904)		
